# Contribution of cats and dogs to SARS-CoV-2 transmission in households

**DOI:** 10.3389/fvets.2023.1151772

**Published:** 2023-07-14

**Authors:** Egil A. J. Fischer, Els M. Broens, Hans S. Kooistra, Myrna M. T. De Rooij, Jan Arend Stegeman, Mart C. M. De Jong

**Affiliations:** ^1^Faculty Veterinary Medicine, Utrecht University, Utrecht, Netherlands; ^2^Department of Quantitative Veterinary Epidemiology, Wageningen University, Wageningen, Netherlands

**Keywords:** final size, zoonosis, multispecies, mathematical model, reproduction number, multilevel transmission, COVID-19, companion animal

## Abstract

**Introduction:**

SARS-CoV-2 is known to jump across species. The occurrence of transmission in households between humans and companion animals has been shown, but the contribution of companion animals to the overall transmission within a household is unknown. The basic reproduction number (*R*_0_) is an important indicator to quantify transmission. For a pathogen with multiple host species, such as SARS-CoV-2, the basic reproduction number needs to be calculated from the partial reproduction numbers for each combination of host species.

**Method:**

In this study, the basic and partial reproduction numbers for SARS-CoV-2 were estimated by reanalyzing a survey of Dutch households with dogs and cats and minimally one SARS-CoV-2-infected human.

**Results:**

For households with cats, a clear correlation between the number of cats and the basic reproduction number (Spearman's correlation: *p* 0.40, *p*-value: 1.4 × 10^−5^) was identified, while for dogs, the correlation was smaller and not significant (Spearman's correlation: *p* 0.12, *p*-value: 0.21). Partial reproduction numbers from cats or dogs to humans were 0.3 (0.0–2.0) and 0.3 (0.0–3.5) and from humans to cats or dogs were 0.6 (0.4–0.8) and 0.6 (0.4–0.9).

**Discussion:**

Thus, the estimations of within-household transmission indicated the likelihood of transmission from these companion animals to humans and vice versa, but the observational nature of this study limited the ability to establish conclusive evidence. This study's findings support the advice provided during the pandemic to COVID-19 patients to maintain distance from companion animals as a precautionary measure and given the possibility of transmission, although there is an overall relatively limited impact on the pandemic when compared to human-to-human transmission.

## Introduction

Since the beginning of the COVID-19 pandemic, SARS-CoV-2 infections have been reported in captive and domestic animals. Felines in the Bronx Zoo were found to be infected (1), and ~14.7% of cats (*Felix catus L*.) tested seropositive in early 2020 in the Wuhan region (2). It was found that domestic cats are susceptible to infection, and virus reproduction can occur (3). Furthermore, infection of cats by infected humans or other species (i.e., mink) has been reported (4). In later experimental studies, transmission between cats was confirmed (5, 6). A Dutch seroprevalence study including companion animals with unknown SARS-CoV-2 exposure status showed 0.4% of cats and 0.2% of dogs (*Canis lupus familiaris* L.) to be positive for COVID-19 in both ELISA-assays and virus neutralization tests (7). Another survey in the Netherlands with dogs and cats from households with at least one human with a confirmed SARS-CoV-2 infection revealed 20.4% of cats and 17.3% of dogs to be seropositive and/or PCR positive (8). Experiments indicated that transmission between cats in confined spaces can be efficient (9). The epidemiological study on the cat population in Wuhan indicated the reproduction number to be low (1.09), implying the cat-to-cat transmission to be certainly possible but not highly efficient (6, 10). The presence of other endemic coronaviruses and reinfection with these viruses in domestic cats indicates that ongoing horizontal transmission of the coronavirus between domestic cats is not unprecedented (11). In 2021, Shia et al. were the first to report a cat-to-human transmission event of SARS-CoV-2 (12). This important finding indicates that cat-to-human transmission is possible, and given the human-to-cat transmission (2, 8) and the cat-to-cat transmission (5, 6, 9), these animals might contribute to the overall reproduction number in households.

Besides cats, SARS-CoV-2 research has also been conducted on dogs, albeit to a lesser extent. The study by Sit et al. showed that the SARS-CoV-2-positive dogs were most likely infected by humans (13). In one transmission experiment, none of the five contact dogs were infected, while only two out of the five inoculated dogs were seroconverted. No infectious virus was found in the swabs collected from inoculated dogs. The other tissues, including the lungs, were negative (3). To our knowledge, this is the only experiment with dogs. It is, however, not possible to draw an inference on the possibility of transmission between dogs due to the small number of animals in this experiment.

In the Netherlands, ~23% of households own one or more cats, with an average of 1.7 cats per household. For dogs, these numbers are 18% and 1.2, respectively (14). Cats and dogs are likely to have intense contact with their owners, such as licking their faces or sleeping in a bed with them (15). This warrants an investigation of the potential role of cats and dogs in spreading SARS-CoV-2 in households. In this study, we quantified the potential role of cats and dogs in the transmission of SARS-CoV-2 within Dutch households by computing the basic and partial reproduction numbers.

## Materials and methods

### Datasets on humans, dogs, and cats in households

For this study, we made use of datasets obtained by a survey in households with at least one cat or dog and with at least one person who tested positive for SARS-CoV-2 by PCR. Samples were collected between July 2020 and April 2021, and during this period, the α-variant (PANGO lineage b.1.1.7) was dominant in the Netherlands (16). The survey was conducted (8) by recruiting households via the municipal health service. Persons could express their interest in participating by sending an e-mail, and they were then contacted for an appointment. The households were visited by a mobile veterinary clinic for the sampling of the animals, which was available twice per week. Animals that tested PCR-positive underwent a follow-up examination, which occurred 1–3 weeks after the initial visit.

For the modeling performed in this study, we assumed that the final size was reached within these households. This implies no occurrences of new infections due to within-household transmission after the moment of recording the number of cases. This assumption was viewed as realistic, given the generation time of around 5 days (17) for the dominant virus variant at the time in the Netherlands and the delay between health service testing and inclusion in this study.

The survey was conducted amongst 196 households, of which five households had missing data for the number of humans in the household, so these were excluded. Of the 191 remaining households, 95 households had one or more cats and 121 households had one or more dogs. Only households for which the test results of all adult household members and companion animals were available were included for data analysis in this study. The resulting dataset comprised a total of 150 cats, 153 dogs, and 593 humans. Following the assumption that an infection in a household started with one human index case, 123 secondary human cases were found in 87 households, 30 infected cats in 24 households, and 27 infected dogs in 24 households.

These data were analyzed for households with cats and dogs assuming equal transmission (which we will name for convenience “companion animals”), with cats only, and with dogs only. It was not feasible to estimate transmission between cats and dogs due to the low number of 25 households in this study with both cats and dogs.

### Within household transmission

The final size of an outbreak is the number of individuals that have been infected during the entire duration of the outbreak. The number of infected individuals during a household outbreak (i.e., the final size) follows a probability distribution, given the probability of all integer numbers between 1 (index) and all individuals being infected. This so-called final size distribution can be determined based on a stochastic epidemiological SIR model for single and multiple types of individuals (18, 19). Thus, we considered the situation where the infection is introduced by one individual in the household.

### Correlation between R_0_ for humans and animals

First, we estimated the reproduction number of humans only but included the ratio of animals to humans in the analysis as a covariate. For this analysis, only households with at least two humans were used because, otherwise, transmission between humans within the household was not possible. The regression coefficient indicates the effect of increasing the ratio of animals to humans in the household on the basic reproduction number. Additionally, we estimated the basic reproduction number for each household separately and determined a correlation with the ratio of companion animals to humans with a Spearman rank correlation test.

### Estimating partial reproduction numbers for within- and between-species transmission

**Figure 1:**
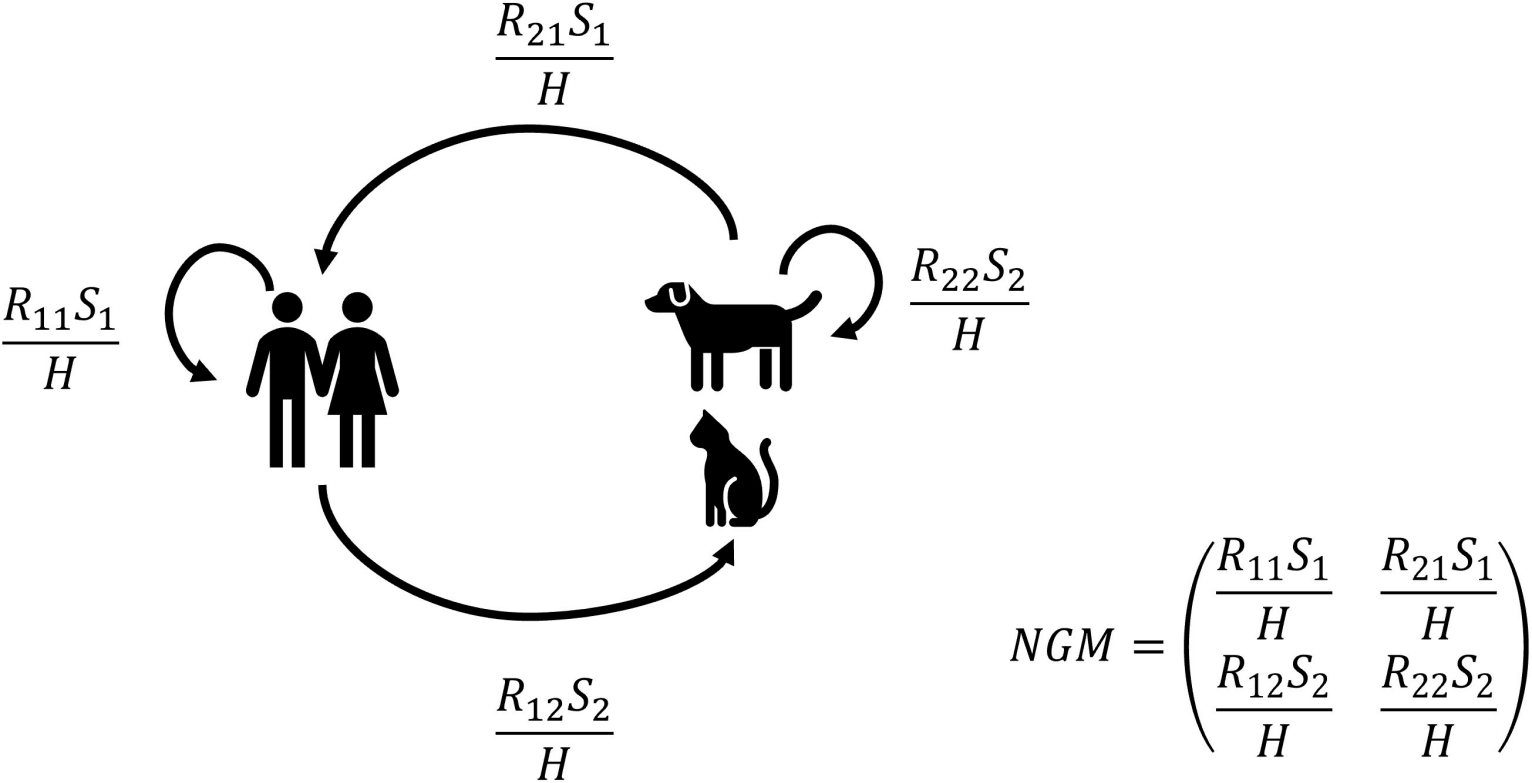
Graphical representation of the transmission model and the next-generation matrix (NGM). Partial reproduction numbers *R*_*ij*_ are the number of new infectious individuals of type j (1 = human or 2 = animal) by an individual of type I (1 = human or 2 = animal). For example, *R*_12_ is the number of new infectious animals (type 2) caused by one infectious human (type 1). The actual number of new infections does also depend on the number of susceptible individuals of a certain type (*S*_1_ or *S*_2_) and the total household size H, i.e., the number of humans in the house.

Next, the partial reproduction numbers (see Figure 1) were calculated for both companion animals, cats only, or dogs only. Partial reproduction numbers *R*_*ij*_ are the number of new infectious individuals of type *j* (1 = human or 2 = animal) produced by an individual of type *i* (1 = human or 2 = animal). For example, *R*_12_ is the number of new infectious animals (type 2) caused by one infectious human (type 1) during its entire infectious period. The actual number of new infections also depends on the number of susceptible individuals of a certain type (*S*_1_ or *S*_2_) and the total household size determined by the total number of humans in the household *H*.

The final size distribution for two types (human and animal) after an introduction by one individual was determined by the number of individuals of each type in the household and these partial reproduction numbers *R*_*ij*_ (18–20). The partial reproduction numbers *R*_*ij*_ are the elements of the next-generation matrix (NGM) that determine the number of new cases of type *j* caused by type *i*. The overall within-household reproduction number *R*_0_ is the largest eigenvalue of the next-generation matrix for a given household composition (i.e., number of humans and companion animals). The equations for the final size distribution and basic reproduction number are given in the Supplementary material and provided as an algorithm in a mathematical notebook.

We used the two-type SIR model to estimate the transmission parameters for a household with humans and companion animals, with humans and cats, or with humans and dogs. For the two-type SIR model, we considered that mixing between humans, companion animals, and human and companion animals is proportionate to the number of humans alone, i.e., the resulting NGM will only contain the ratio of companion animals to humans ($\frac{C}{H}$). See Supplementary material. The basic reproduction number for a household with a specific ratio of companion animals to humans was then calculated by the following Equation (20):


(1) $$\begin{array}{l} R_{0}=\frac{1}{2}\left(R_{11}+R_{22}\frac{C}{H}+\sqrt{{\left(R_{11}-\frac{C}{H}R_{22}\right)}^{2}+4R_{12}R_{21}\frac{C}{H}}\right)\; \end{array}$$

Parameter estimation for the within-household model was obtained by maximum likelihood estimation based on the final size distribution. The confidence intervals for the partial reproduction numbers *R*_*ij*_ (the elements of the NGM) were derived using profile likelihood methods. The confidence interval for the overall reproduction number *R*_0_ were obtained using a bootstrapping procedure.

### Scenarios

To calculate the effect of reducing contact with animals and between animals, we adapted our model to include a proportionate decrease in time spent by animals in contact with other household members, for example, because these are outdoors. To enable the calculation of differences in time spent in contact with infected families, we distinguished between the number of contacts made by an infectious animal (ω_*I*_) and susceptible animals (ω_*S*_). Although, in practice, this is often impossible, it allowed us to study the effect of reducing spread by preventing transmission to animals and by preventing transmission from animals. The transmission matrix within the household **R** is as follows:


(2) $$\begin{array}{l} \text{R}=\left(\begin{array}{cc} \;R_{11} & \left(1-\omega_{I}\right)R_{21}\\ \left(1-\omega_{S}\right)R_{12} & (1-\omega_{I})(1-\omega_{S})R_{22} \end{array}\right) \end{array}$$

With the heterogeneity within the household model, we calculated the mean final sizes for different household compositions when the infection is introduced by an infected cat or by an infected human. Four different scenarios are shown with cats either spending their time completely in contact with household members, having no contact with susceptible animals, having no contact with infected animals, or having no contact with animals at all.

### Software

All data processing and calculations were conducted using Wolfram Mathematica version 12.0. The Mathematica notebook can be found in the Supplementary material.

## Results

### Estimation of within-household transmission

#### Human-to-human with cats and dogs, cats, or dogs as a covariate

First, we estimated the reproduction number of humans without considering animals as a different type in the transmission model but included them in the analysis as a covariate. For this analysis, only households with at least two humans were used because, otherwise, transmission within the household was not possible. The overall human-to-human reproduction number *R*_0_ was 1.17 (0.92–1.47) in this dataset.

The individual household reproduction numbers were calculated, and these were positively correlated with the number of animals (Spearman ρ 0.28, *p*-value 1.8 x 10^−5^) and cats (Spearman ρ 0.40, *p*-value 1.4 x 10^−5^) but not with dogs (Spearman ρ 0.12, *p*-value 0.21). Including the human-to-animal ratio and human-to-cat ratio in the estimation of the basic reproduction number *R*_0_ improved the fit of the model and again showed an increase in *R*_0_ with cats per human. In contrast, the human-to-dog ratio did not improve the model fit (Table 1).

**Table 1:** Reproduction number for human-to-human transmission with the effect of animal-to-human ratio as a covariate.

**Model**	**R_0_**	**95%-CI**	**Effect of animal/human-ratio**	**95%-CI**	**AIC**
Baseline	1.17	(0.93–1.47)			363
Companion animals	0.89	(0.57–1.34)	1.97	(0.18–4.79)	355
Cats	0.91	(0.57–1.39)	1.19	(-0.05–2.97)	358
Dogs	1.10	(0.69–1.66)	0.33	(-0.15–2.19)	364

The effect of animals should be interpreted as an increase in reproduction numbers in a household with an increase of one animal per human. R0, reproduction number, 95%-CI, 95%-confidence interval, and AIC, Aikaike's information criterion.

The household sizes of cat (mean 2.3, se 0.1) and dog owners (mean 2.4, se 0.1) did not differ (*t*-test −0.75, *p* = 0.45).

#### Human and companion animals as separate types in transmission modeling

In total, 191 households with complete records containing one or more companion animals, i.e., cats, dogs, or both, were included in the estimation procedure. In these households, 314 out of 593 humans and 56 out of 303 dogs and cats tested positive, respectively.

This gives a partial reproduction number for human-to-human transmission of 1.19 (0.90–1.44) and for human-to-companion animal transmission of 0.63 (0.42–0.77). The estimates on transmission from companion animals to humans and between companion animals have a higher level of uncertainty due to the low numbers of infected companion animals and the assumption that, for each household, a human introduced SARS-CoV-2. The companion animal-to-human transmission was 0.39, for which the lower limit remains undetermined, indicating that it was very close to zero, and the upper limit was 2.15, and companion animal-to-companion animal transmission was 0 with an upper limit of 0.27. The lower limit of an estimate equals the point estimate if the point estimate is zero. The results are summarized in Table 2.

**Table 2:** Estimates for transmission between companion animals and humans. The partial reproduction numbers *R*_*ij*_ have subscripts indicating transmission of *i* to *j*; 1 = human and 2 = animal (companion animal, cat, or dog).

	**Human and companion animal**		**Human and cat**		**Human and dog**	
						
*R*_11_	1.19	(0.90–1.44)	1.26	(0.92–1.49)	1.11	(0.88–1.44)
*R*_21_	0.39	(0.00–2.15)	0.30	(0.00–2.02)	0.30	(0.00–3.48)
*R*_12_	0.63	(0.42–0.77)	0.56	(0.36–0.78)	0.57	(0.41–0.86)
*R*_22_	0.00	(0.00–0.27)	0.00	(0.00–0.48)	0.00	(0.00–1.26)

**Figure 2:**
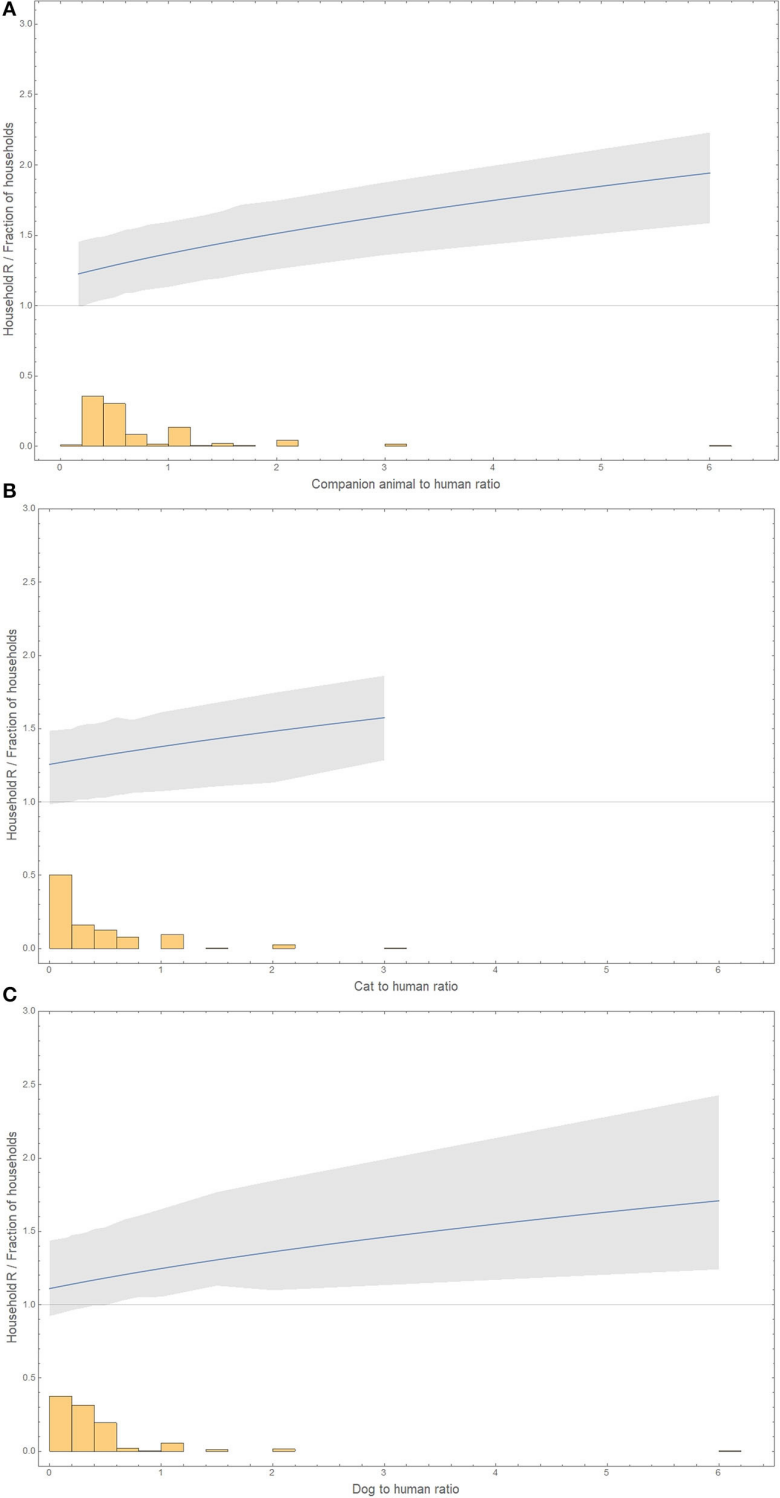
Animal-to-human ratio distribution (histogram) and the basic reproduction number for these household compositions (line and bootstrap 95% confidence interval as a gray area). **(A)** Companion animals. **(B)** Only cats. **(C)** Only dogs. The x-axis has for dogs or companion animals to humans a maximum of 6, and for cats to humans, the maximum was 3. The y-axis represents either the household's basic reproduction number or the fraction of households with a certain ratio of animals to humans. The basic reproduction number is shown only in the range of observed values of animal-to-human ratios.

The basic reproduction number in a household increases with the ratio of companion animals to humans, from 1.19 (0.90–1.44) in the absence of companion animals to 1.94 (1.59–2.23) with six companion animals to one human (Figure 2).

#### Humans and cats as separate types in transmission modeling

In total, 191 households with complete records, of which 91 contained one or more cats, were included in the estimation procedure. In these households, 314 out of 592 humans and 30 out of 150 cats tested positive. We did not consider dogs to be infectious in these analyses.

This resulted in a partial reproduction number for human-to-human transmission of 1.26 (0.92–1.49) and for human-to-cat of 0.56 (0.36–0.78). The transmission estimates from cats to humans and between cats have a higher level of uncertainty due to the low numbers of infected cats and the assumption that, for each household, a human introduced SARS-CoV-2. The cat-to-human transmission was 0.30 (0.00–2.02), and the cat-to-cat transmission was zero, with an upper limit of 0.63. The lower limit of an estimate equals the point estimate if the point estimate is zero. The results are summarized in Table 2.

The basic reproduction number increases with the ratio of cats to humans, from 1.26 in the absence of cats to 1.58 (1.29–1.86) with three cats to one human (Figure 2).

#### Humans and dogs as separate types of transmission modeling

In total, 110 households with complete records containing one or more dogs were included in the estimation procedure. In these households, 186 out of 378 humans and 26 out of 134 dogs tested positive, respectively. We did not consider cats to be infectious in these analyses.

This resulted in a partial reproduction number for humans-to-humans of 1.1 (0.88–1.44) and the human-to-dog of 0.57 (0.41–0.86). The estimates on transmission from dogs to humans and between dogs have a higher level of uncertainty due to the low numbers of infected dogs and the assumption that, for each household, a human introduced SARS-CoV-2. The dog-to-human transmission was 0.33, for which the lower limit was 0.00, the upper limit was 3.38, and the dog-to-dog transmission was 0, with an upper limit of 1.26. The lower limit of an estimate equals the point estimate if the point estimate is zero. The results are summarized in Table 2.

The basic reproduction number increases with the ratio of dogs to humans from 1.11 in the absence of dogs to 1.71 (1.24–2.43) with six dogs to one human (Figure 2).

### Scenarios

#### Within-household

**Figure 3:**
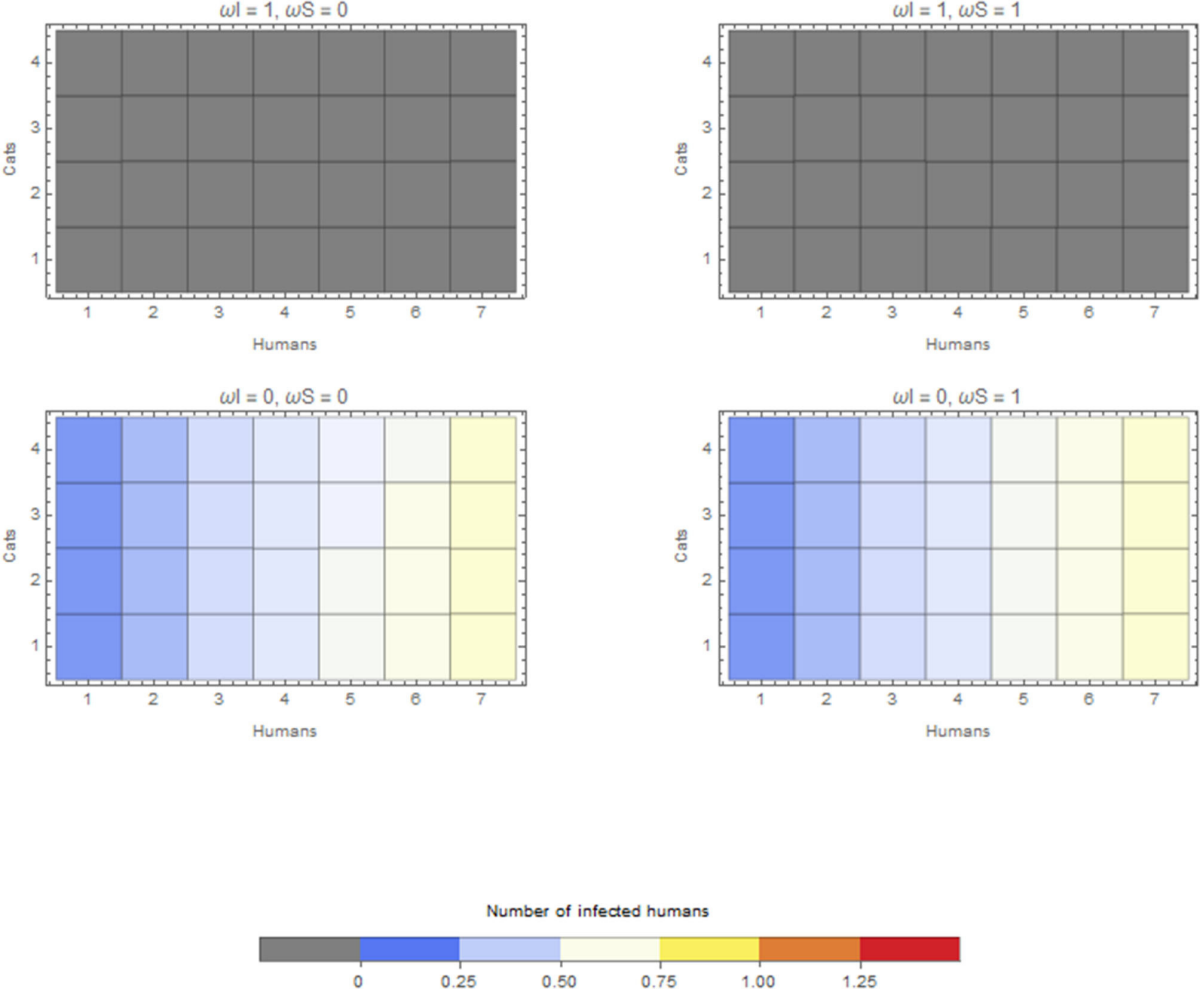
Average final size of infected humans when the infection is introduced by a cat for extreme values of ω_*I*_ and ω_*S*_. Cats that do not go outside ω = 0 or are always outside ω = 1.

The within-household outbreak size in relation to the duration of time spent in the household by companion animals is presented here for cats only. Cats have the largest effect, given the estimates above. A similar pattern was found for companion animals and dogs. In the scenario considering a cat as the index case in a household, if this infectious cat does not spend any time within the household (ω_*I*_ = 1), this cat will not cause an outbreak in the household (top two panels of Figure 3). If this index case cat is kept within the household all the time (ω_*I*_ = 0), the final size depends on the number of cats in the household and depends on whether these susceptible cats are kept inside (bottom two panels of Figure 3). Keeping both susceptible and infectious cats inside (left bottom panel of Figure 3) causes the largest within-household outbreaks. However, these differences are subtle.

**Figure 4:**
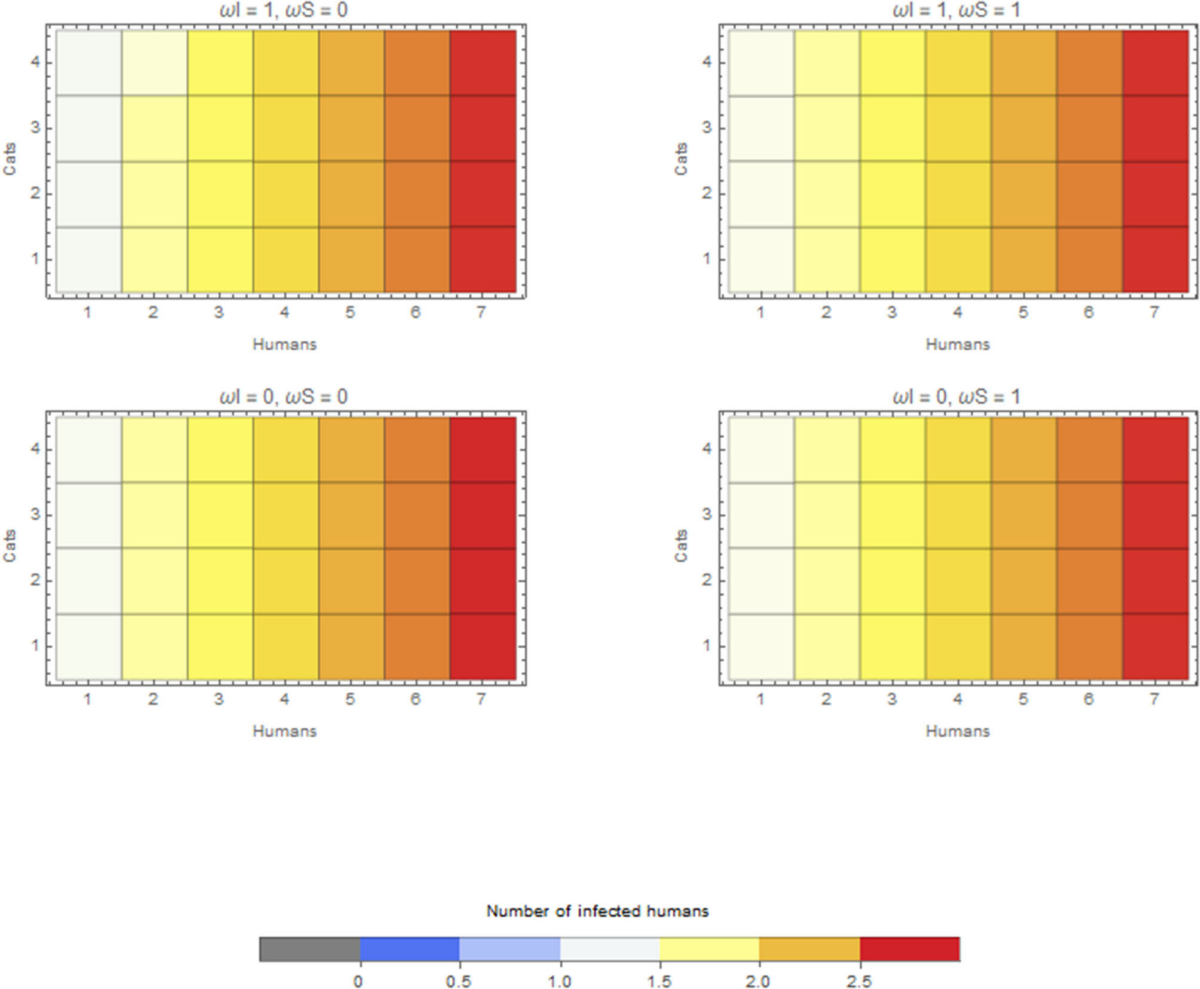
Average final size when the infection is introduced by a human for extreme values of ω_*I*_ and ω_*S*_. Cats do not go outside ω = 0, or cats are always outside ω = 1.

In the scenario with a human as the index case in a household, the median final size of the outbreak is 2.17, irrespective of whether cats are kept inside the house or outside. The maximum final size is, however, slightly different, that is, 2.78, when cats are kept inside the house and 2.75 when kept outside. The overall effect is thus limited compared to the role of humans regarding within-household transmission (Figure 4).

## Discussion

The findings regarding within-household transmission showed a likely but not conclusive indication of SARS-CoV-2 transmission from companion animals to humans, especially for cats. The ratio of cats and companion animals was associated with an increase in the reproduction number in a household, which was not found for dogs alone. For both species, a non-zero estimated value was found for animal-to-human transmission, but this could not be distinguished statistically from zero.

Although our findings may indicate a potential role for companion animals and, in particular, cats in the within-household transmission of SARS-CoV-2, some caution in the interpretation is required. We did not consider other confounding factors like behavior, housing conditions, or the age of the owner, which could be related to increased transmission and ownership of cats or dogs, and the number of companion animals in a household. Still, given the evidence for spill-over between species of SARS-CoV-2 (2, 4, 7, 12), a role in transmission is conceivable, and these analyses provide additional evidence and quantification of the partial reproduction numbers.

Both the results of analyses of the correlation between *R*_0_ when only considering humans as hosts and the ratio of companion animals and the estimates for the model with both transmissions to and from companion animals indicate stronger evidence for cats having a potential role in transmission than dogs. For both species, we found non-zero estimates for transmission from animal to humans, but the overall uncertainty for dogs was greater. Additionally, we found a positive correlation for cats in the overall *R*_0_ when only considering humans as hosts, but not for dogs. *R*_0_ does not depend on the household size; thus, an increase in *R*_0_ with more animals per human suggests additional transmission by these animals, or, as stated above, it can be due to a confounding factor. Furthermore, the average number of cats per household is higher, which increases the potential transmission to and from these animals. The role of dogs can, however, not be disregarded (yet), given the current limited knowledge. It is worth noting the overall limited role of cats and dogs compared to human-to-human transmission in light of the course of the pandemic.

The reproduction rate of both cats and dogs in experimental settings is highly uncertain. Inferring *R*_0_ the data of Bosco-Lauth et al. (9) gives an estimate of 0.37 to infinity. Experiments by Gehrards et al. (6) obtained a basic reproduction number of 2.50 (0.97–5.15). Our results on households are lower than those experiments and bounded by 0. For stray cats in Wuhan with no known contact with humans, the basic reproduction number was estimated to be ~1.1 (10). In the sensitivity analysis, when the cat-to-cat reproduction number was fixed at a value of 1.1, no relevant change in parameters was observed. When both the cat-to-cat and human-to-human reproduction numbers were fixed at values of 1.1 and 1.4, respectively, the cat-to-human transmission was estimated to be zero. However, it is important to note that there was an upper limit of 1.63 in the confidence interval (see Supplementary material). This shows that our results cannot unequivocally prove cat-to-human transmission in these households nor disprove this route. Experimental studies provide evidence that cats reproduce the virus and can transmit it to other cats and, thus, most probably also to other mammals (6, 9). Animal-to-human transmission of SARS-CoV-2 has been observed in farmed mink and farm workers, showing the potential for transmission from animals to humans (21). Furthermore, the report of a cat-to-human transmission event in Thailand makes this route more probable (12).

An experimental study with dogs did not observe transmission between dogs. This could indicate that dogs might not be able to transmit the infection, but this study was too small in size to draw such conclusions (3). Only two out of five inoculated dogs did seroconvert, and solely two susceptible dogs were added as contacts. Consequently, owing to this lack of power, the confidence interval on the reproduction number was wide, with an upper limit of 44.

In the Netherlands, the number of people per household (the household size) is typically relatively small (on average 2.13). We had not considered other living conditions with intense contacts, such as a student housing with shared sanitary and kitchen facilities. Most domestic cats live in households alone or with one other cats (14), so there cannot be a sustained endemic within a household. It should be taken into consideration that we did not include feral cats or catteries. These larger groups of cats could become reservoirs from which reintroduction of the virus is possible. In Wuhan, China, a high prevalence of cats shows the potential for a reservoir among cats found on the streets (2). However, this has not been identified in the Netherlands to date based on the low seroprevalence detected in shelter cats (22). Quantification of the likelihood of a reservoir in feral cats requires another modeling approach, including a thorough quantification of the interaction between different colonies of feral cats, between catteries, and between animal shelters. Moreover, dogs tend to be kept alone in households. The population of feral or semi-feral dogs and, of course, shelters could also be potential populations for sustained transmission amongst dogs.

In contrast to feral cats (23), little is known about the interaction between domestic cats related to infectious diseases outside their own households. Interactions between cats from other households could occur during fights or by transmission via the environment in overlapping territories. Dog-to-dog transmission outside the household can occur when walking the dog or in shared airing areas. Transmission from companion animals to humans or human-to-companion animals could occur via the same pathway via the environment or by petting. Furthermore, feeding feral or neighboring cats might be a way in which cats are stimulated to visit more than one household. This can potentially contribute to between-household transmission (see Supplementary material), but to determine this with any certainty, data on between-household transmission are required.

Although vaccination coverage in many countries is reaching the required level (https://covid19.who.int/table), the virus can still spread among the vaccinated population due to waning immunity or new variants (24). It is, therefore, warranted to keep track of SARS-CoV-2 susceptible companion animals in the household when considering the epidemiology of SARS-CoV-2 in humans. Close contact with companion animals by SARS-CoV-2-positive humans increases the probability of this animal becoming infected, but if animals are kept inside for a sufficiently long period, this will mitigate the risk of animals acting as vectors between households (see Supplementary material). In the Netherlands, people with a SARS-CoV-2 diagnosis/suspicion are advised to keep their distance from companion animals, which is also supported by this study, although it should be taken into consideration that the overall contribution of companion animals is limited. Moreover, avoiding contact with companion animals from other households, even when visiting these households, can be viewed as a general method to reduce the impact of companion animals acting as potential vectors for the transmission of infectious diseases between households.

## Data availability statement

The original contributions presented in the study are included in the article/Supplementary material, further inquiries can be directed to the corresponding author.

## Ethics statement

Ethical review and approval was not required for the study on human participants in accordance with the local legislation and institutional requirements. Written informed consent to participate in this study was provided by the participants' legal guardian/next of kin. Ethical review and approval was not required for the animal study because this study was conducted on previously obtained data. Sampling of the animals was approved by the Animal Care and Ethics Committee of Utrecht University, in accordance with the Dutch law on experimental animals (approval number AVD1080020209666). Written informed consent was obtained from the owners for the participation of their animals in this study.

## Author contributions

EF and MCMD had an equal contribution to the development and application the mathematical models. MCMD and JS initiated the study. EB, MMTD, and HK provided the data and discussed the interpretation of the outcomes of mathematical models. All authors contributed in writing the manuscript.
