# Supplementary material for: Contribution of cats and dogs to SARS-CoV-2 transmission in households
Source: Front Vet Sci. 2023 Jul 14;10:1151772. doi: 10.3389/fvets.2023.1151772 (PMC10375487; doi:10.3389/fvets.2023.1151772)
Supplement: Supplementary file 1 [file Data_Sheet_1.PDF]

Supplementary information with "Contribution of cats and dogs to SARS-CoV-2 transmission in households"

E.A.J. Fischer*[,1§], E. Broens[1], H.S. Kooistra[1], M.M.T. de Rooij[1], J.A. Stegeman[1], M.C.M. De Jong*[,2]

1. Faculty Veterinary Medicine, Utrecht University, Yalelaan 7, 3584 CL, Utrecht, The Netherlands

2. Department of Quantitative Veterinary Epidemiology, Wageningen University, 6700 AH, Wageningen, The Netherlands

*These authors contributed equally to the study

§Corresponding author: e.a.j.fischer@uu.nl

Table of Contents

1. Within-household model details 2
  1.1. Derivation of within-household reproduction number 2
  1.2. Equations and estimation procedure for the final size distribution 2
  1.3. Maximum likelihood estimation 3
2. Sensitivity analysis for within-household estimation 5
  2.1. Method and data 5
  2.2. Result 5
3. Between-household model 7
  3.1. Between household model 7
  3.2. Equations of the between household model 7
  3.3. Input data 8
    3.3.1. Household size and companion animal distribution in the general population 8
    3.3.2. Distribution of cases 9
    3.3.3. Household size distribution 9
    3.3.4. Cat distribution 10
    3.3.5. Parameterization between-household transmission and scenarios 10
  3.4. Results Between household transmission 10
4. References 11

1. Within-household model details

Calculations were done in Mathematica 12. The notebook can be found in the supplementary materials.

1.1. Derivation of within-household reproduction number

For the within-household transmission model we define the reproduction number for host type i to host type j. We assume that the mixing is proportionate to humans only (see main text), such that the number of new infections in the next generation is:

$$k_{ij} = S_i \frac{I_j}{H} R_{ij}$$

in which H is the size of the human population and S_i the number of susceptibles of type i.

The reason for doing so is that the derivation of the divisor for the frequency-dependent model comes from the space in which hosts interact (1). For hosts of the same species heterogeneity among host will not affect the contacts especially when differences (e.g. vaccinated or not) are not known to the individuals. Thus with increasing number of hosts the probability to encounter a specific other individual host will decrease, but the number of contacts in total remains equal and thus the R is independent of population size. However, for transmission of hosts of different species this reasoning does not apply. For example for the vector and host models contacts between the human hosts do not normally decrease because of the number of mosquito vectors. The same we therefor assumed to apply to companion animals in a household.

From this we obtain a next generation matrix NGM:

$$NGM = \begin{pmatrix} \frac{R_{11}S_1}{H} & \frac{R_{21}S_1}{H} \\ \frac{R_{12}S_2}{H} & \frac{R_{22}S_2}{H} \end{pmatrix}$$

In the disease-free state the numbers of susceptibles equal the species' entire population thus $S_1 = H$ and $S_2 = C$ (for companion animal population size) and the largest eigenvalue of this NGM provides the basic reproduction number (2) :

$$R_0 = \frac{1}{2}\left(R_{11} + R_{22}\frac{C}{H} + \sqrt{(R_{11} - \frac{C}{H} R_{22})^2 \quad + 4\, R_{12}\, R_{21}\frac{C}{H}}\right)$$

1.2. Equations and estimation procedure for the final size distribution

The within-household model is a stochastic discrete SIR model in which individuals are distinguished as susceptible, infectious or recovered. We define two types of individuals in households, humans (type 1) and cats (type 2). The number of individuals in a household is considered to be constant (N_i) during the household outbreak.

The initial state of the model is given by the number of recipient (X) and infected (Y) humans (type 1) and cats/dogs (type 2) in a household: $\{X_1(0), Y_1(0), X_2(0), Y_2(0)\}$. Assuming that none of the humans or cats is immune (R) at the start. If needed the R individuals have to be added to the population totals. The total number of individuals of type i is thus $N_i = X_i + Y_i$. A state is left either by infection of a human or cat or by recovery of a human or cat.

The probability of being in a certain state $\{X_1, Y_1, X_2, Y_2\}$ is given by:

$$P(\{X_1,Y_1,X_2,Y_2\}) = P(\{X_1,Y_1+1,X_2,Y_2\})\,\frac{(Y_1+1)}{(Y_1+1)+Y_2}\,\mathcal{L}\big(z(X_1,X_2,R_{11},R_{12})\big) + P(\{X_1,Y_1,X_2,Y_2+1\})\,\frac{(Y_2+1)}{Y_1+(Y_2+1)}\,\mathcal{L}\big(z(X_2,X_1,R_{22},R_{21})\big) + P(\{X_1+1,Y_1-1,X_2,Y_2\})\sum_{k=1}^{2}\frac{(Y_1-1)}{(Y_1-1)+Y_2}\left(1-\mathcal{L}\left(z\big((X_1+1),X_2,R_{k1}\big)\right)\frac{z(X_1+1,R_{j1})}{z(X_1+1,R_{k1})+z(X_2,R_{k2})}\right) + P(\{X_1,Y_1,X_2+1,Y_2-1\})\sum_{k=1}^{2}\frac{(Y_2-1)}{Y_1+(Y_2-1)}\left(1-\mathcal{L}\big(z(X_1,X_2+1,R_{k2})\big)\frac{z(X_2+1,R_{k2})}{z(X_1,R_{k1})+z(X_2+1,R_{k2})}\right) \qquad \text{Eq. S1}$$

Ball (3) showed in his seminal paper that the expected probability of recovery before a new infection occurs can be obtained by integrating the probability of no infection ($e^{-\phi_{ij}\frac{X_i}{N_i}\tau}$) over all times when these times have probability density function $f(\tau)$:

$$E(p_{rec}) = \int_o^\infty f(\tau)\, e^{-\phi_{ij}\frac{X_i}{N_i}\tau}\, d\tau$$

which is the Laplace transform of $f(\tau)$ ($\mathcal{L}(z) = \int_o^\infty f(\tau)\, e^{-z\,\tau} d\tau$), and $z(X_i,X_j,R_{ii},R_{ij}) = \frac{R_{ii}X_i+R_{ij}X_j}{w_i\,N_i+w_jN_j}$ or $z(X_i,R_{ij}) = \frac{R_{ij}X_i}{w_i\,N_i+w_jN_j}$. The Laplace transform for commonly used infectious periods (fixed, exponentially distributed or gamma distributed) are known. Without loss of generality the infectious parameters can be scaled to an average infectious period of 1 (4). Mixing is proportionate to the types *i* and j individuals given w_i and w_j. In this study we used $w_i = 1$ and $w_j = 0$, because we observed an increasing reproduction number with the ratio of animals to humans.

With initial condition $P(\{X_1(0),Y_1(0),X_2(0),Y_2(0)\}) = 1$ and the final size distribution i.e. the distribution when the outbreak has ended $\{Y_1 = 0, Y_2 = 0\}$ and can be obtained by solving for all $P(\{\mathrm{k}_1,0,\mathrm{k}_2,0\})$ for all combinations of $\mathrm{k1} = 0,1 \ldots \mathrm{X1}(0)$ and $\mathrm{k2} = 0,1,..\mathrm{X2}(0)$. This can be done by calculating all P({k1,w1,2,w2}) in the right order so that at any point in the calculation all the values of P at the RHS of the recursion formula have already been calculated.

In the Mathematica Algorithm this is done by the Mathematica software that can recursively calculate all values as long as the recursion is programmed correctly and does not exceed the maximum recursion depth (which can be set). In addition to the recursion formula given in S1 the formula for the initial condition and zeroes for values outside the attainable values have to be added.

1.3. Maximum likelihood estimation

If we define the probability of a certain outcome m is $\{X_{1,m},X_{2,m}\}$ given the basic reproduction numbers $(R_{11},R_{12},R_{21},R_{22})$ and starting situation $\{X_{1,m}(0),Y_{1,m}(0),X_{2,m}(0),Y_{2,m}(0)\}$ we can obtain the maximum likelihood estimation of the reproduction numbers by maximizing

$$L(R_{11},R_{12},R_{21},R_{22}) = \prod_{m=1}^{M} P\big(\{X_{1,m},X_{2,m}\}\ |\{X_{1,m}(0),Y_{1,m}(0),X_{2,m}(0),Y_{2,m}(0)\}\ ,R_{11},R_{12},R_{21},R_{22}\big)$$

The confidence intervals for the reproduction numbers were obtained by determining the profile likelihood constrained by $(R_{11} \geq 0, R_{12} \geq 0, R_{21} \geq 0, R_{22} \geq 0)$. The confidence intervals for the

overall R_0 within households was obtained by bootstrapping 1000 samples. For each ratio of animals to humans we calculated the R_0 and obtained the 95% interval for these ratios.

2. Sensitivity analysis for within-household estimation

2.1. Method and data

We performed sensitivity analysis on the estimation of the within-household reproduction numbers. We estimated the human to animal and animal to human transmission while fixing the intra-type reproduction numbers (R_{11} = 1.4 and R_{22} = 1.1). Furthermore we estimated the reproduction numbers by adding the data from another Dutch study (5) assuming that these households do not have cats or dogs to investigate the potential selection bias of the survey by only including households with a companion animal. The numbers for the analysis in which extra households were added, should however be interpreted as sensitivity analysis because of uncertainty of whether or not these HH had cats or dogs and not as actual quantification of these parameters.

2.2. Result

Adding the 55 Dutch households for which we assumed no companion animals were present (5) slightly changed the estimation by increasing the human to human transmission, but hardly changed the values for the animals. The human-to-human reproduction number might be overestimated, and the numbers to and from animals are an underestimation due to the fact that of the added households 18% are expected to own dogs and 23% are expected to own cats. The addition does, however, show that the error made by only including households with animals is minor.

Fixing the reproduction numbers quantitatively changed the estimates, but only when we fixed the human-to-human transmission to a value higher than estimated from the baseline data (i.e. 1.4) the companion animal, cat or dog to human transmission had a maximum likelihood estimator of 0.00, but with an upper limit high above zero.

	Baseline		*Households added*		*Fix R_{22}*		*Fixed R_{11} and R_{22}*	
		95%-CI		95%-CI		95%-CI		95%-CI
	**Companion animals**							
R_{11}	1.19	(0.90 – 1.44)	1.39	(1.07 – 1.56)	1.12	(0.85 – 1.47)	*1.40*	
R_{21}	0.39	(0.00– 2.15)	0.32	(0.00 – 1.55)	0.64	(0.00 – 2.28)	0.08	(0.00 -1.31)
R_{12}	0.63	(0.42 – 0.77)	0.64	(0.44 – 0.78)	0.57	(0.47 – 0.76)	0.59	(0.47 – 0.77)
R_{22}	0.00	(0.00 -0.27)	0.00	(0.00 – 0.27)	*1.10*		*1.10*	
	**Cats**							
R_{11}	1.26	(0.92 – 1.49)	1.47	(1.09 – 1.75)	1.18	(0.92 – 1.50)	*1.40*	
R_{21}	0.30	(0.00 – 2.02)	0.36	(0.00 – 4.14)	0.37	(0.00 – 2.28)	0.00	(0.00 -1.63)
R_{12}	0.56	(0.36 – 0.78)	0.66	(0.47 – 0.95)	0.58	(0.43 – 0.85)	0.59	(0.44 – 0.87)
R_{22}	0.00	(0.00 – 0.48)	0.00	(0.00 -0.66)	*1.10*	-	*1.10*	
	**Dogs**							
R_{11}	1.11	(0.88 – 1.44)	1.22	(1.05 – 1.59)	1.11	(0.86 -1. 43)	*1.40*	
R_{21}	0.30	(0.00 – 3.48)	0.25	(0.00 – 3.01)	0.81	(0.00 – 3.55)	0.24	(0.00 -2.28)
R_{12}	0.57	(0.41 – 0.86)	0.57	(0.42 – 1.04)	0.66	(0.46 – 0.98)	0.68	(0.47 – 1.02)
R_{22}	0.00	(0.00 – 1.26)	0.00	(0.00 – 1.85)	*1.10*		*1.10*	

Table S- 1 Estimates for transmission of companion animals and humans. The baseline is estimated from the data of the Dutch survey and the households added is based on the Dutch survey and the data of (5) or by estimation with a fixed value for the reproduction number R_{22} or R_{11} and R_{22}. R_{11} is human-to-human, R_{21} is cat to human, R_{12} is human to cat and R_{22} is cat to cat.

3. Between-household model

SARS-CoV-2 in humans and companion animals is an infection spreading within and between households. Such a population constructed of subpopulations is called a metapopulation. By transmission between members of different households the reproduction number of the infection for a metapopulation can be calculated (6,7). This reproduction number has the threshold property that above one the infection can spread, while below one it will go extinct.

We analysed the impact of between-household transmission, but only for cats. The results for dogs would be similar.

3.1. Between household model

We assume that a household outbreak and thus its final size is caused by only one introduction by either a human or animal in that household. The reproduction number in a household-structured population R^* is defined as the number of new infectious individuals produced within other households due to infection by an infector outside the own household. In other words, it counts all infections in household as if they were produced by the infector from another household that caused the first infection in that household (6,7). In the papers cited and in (8) it is shown that this is the correct reproduction ratio between individuals for such a household-structured population.

Infections between households are determined by the global reproduction rates R_{ij}^{G}, which is defined as the number of new primary infections of type *j* caused by an infector of type *i* outside the own household during its entire infectious period.

The total number of new infections of type *j* caused by an individual of type *i* the sum over the average number of infected of type *j* in a household infected by an individual *i.* The number will be called R_{ij}^{*}.The probability of infecting an individual of type *i* in a household of a certain composition (number of type 1 and 2) is determined by the household size distribution $h(N_1,N_2)$ that is to say by the fraction of all individuals of type *i* living in a household of size (N_1,N_2).

For this system we define a NGM with each of the four elements R_{ij}^{*} (2). The largest eigenvalue of the next generation matrix is the reproduction ratio in a community of household R^* (6,7). The normalized right eigenvector of this matrix gives the stable distribution of infection among humans and cats. The single elements of the NGM R_{ij}^{*} determine the number of infections of type *i* to type *j* .

3.2. Equations of the between household model

The expected number R^* that a typical (average) infectious individual infects in a household structured community is defined by attributing all infections caused *globally* (i.e. in the whole metapopulation of households) and all secondary household infections caused by this global infection to the infector, and not the infections in the own household. Attribution of infections to infectors in this way means that we assume that households are infected only by one global infector. This definition also means that human infections in a household caused by a global infection of a cat are attributed to the global infector, the cat in this case.

The partial reproduction number R_{ij}^{*} is thus defined as the number of infections of type j by infector type i in other households.

$$R_{ij}^{*} = R_G(i,j) \sum_i \sum_j \mu_{N_i,N_j} \frac{N_j \, \pi_{N_i,N_j}}{\sum_j \sum_i N_j \, \pi_{N_i,N_j}}$$

in which $R_G(i,j)$ is the global reproduction number between type *i* and *j*, μ_{ij} the average number of infected individuals of type *j* when introduced in the household by infector of type *i* with *i* and *j* individuals in the household. Furthermore π_{N_i,N_j} is the fraction of household with N_i and N_j individuals in the total household population, thus $\frac{N_j \pi_{N_i,N_j}}{\sum_j \sum_i N_j \pi_{N_i,N_j}}$ is the fraction of individual of type *j* living in a household with N_j and N_i individuals of both types.

The average number of infected individuals of type *j* when introduced in the household by infector of type *i* with *i* and *j* individuals in the household, μ_{ij}, is calculated by the final size distribution:

$$\mu_{N_i,N_j} = \begin{cases} 1 + \sum_{k}^{N_j-1} \sum_{l}^{N_i} k\, P_{k,l}\left(\{k,0,l,0\} \middle| \{N_j-1,1,N_i,0\}\right), & i = j \\ \sum_{k}^{N_j} \sum_{l}^{N_i-1} k\, P_{k,l}\left(\{k,0,l,0\} \middle| \{N_j,0,N_i-1,1\}\right), & i \neq j \end{cases}$$

The four partial reproduction numbers define the next generation matrix:

$$NGM = \begin{pmatrix} R_{11}^* & R_{21}^* \\ R_{12}^* & R_{22}^* \end{pmatrix}$$

from which the reproduction number R^* can be obtained by calculating the largest eigenvalue (2,6,7) for assumption that numbers of the two hosts count equally in the divisor:

$$R^* = \frac{1}{2}\left(R_{11}^* + R_{22}^* + \sqrt{(R_{11}^* + R_{22}^*)^2 - 4(R_{11}^* R_{22}^* + R_{12}^* R_{21}^*)}\right)$$

3.3. Input data

3.3.1. Household size and companion animal distribution in the general population

The overall household size distribution in the Netherlands was obtained from the Dutch bureau of statistics (https://opendata.cbs.nl/#/CBS/en/dataset/82905ENG/table?dl=48C2D) for the year 2019. The data categorize households in size 1 to 4 and larger than 5. In the calculations the category larger than 5 was split up in 50% size 5, 25% size 6 and 25% size 7.

Of all households 23% own one or more cats with an average of 1.7 per household (9). We modelled the distribution over households as owning 1 cat plus a binomial distributed (n=3, p=0.7/3) number of cats ranging from 0 to 3 additional cats per household. This distribution was used for the model of between-household transmission.

3.3.2. Distribution of cases

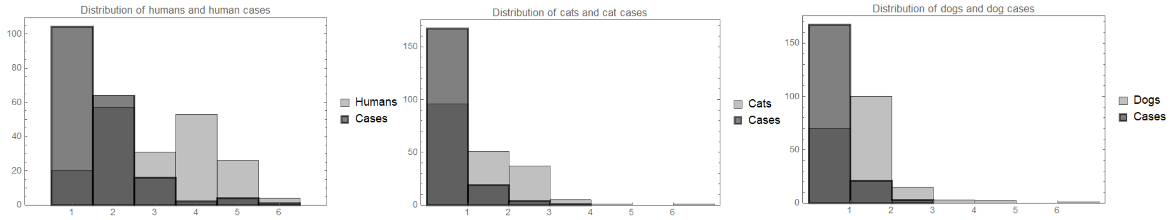

Figure 1 Distribution of household sizes and infected humans and companion animals in the survey.

3.3.3. Household size distribution

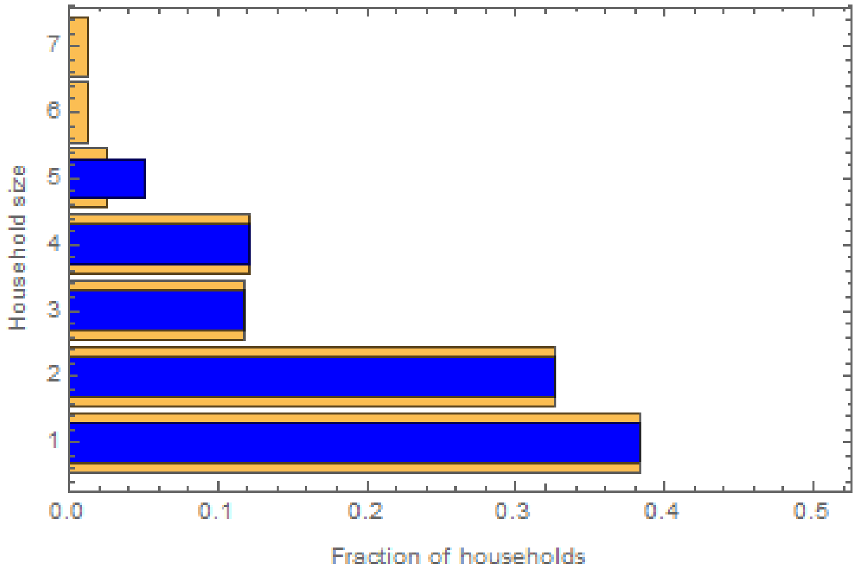

Figure 2 Household size distribution used in the calculations (orange bars). The census data is given as blue bars (https://opendata.cbs.nl/#/CBS/en/dataset/82905ENG/table?dl=48C2D). Census data category >5 has been split into households of size 5, 6 and 7 in a 2:1:1 ratio.

3.3.4. Cat distribution

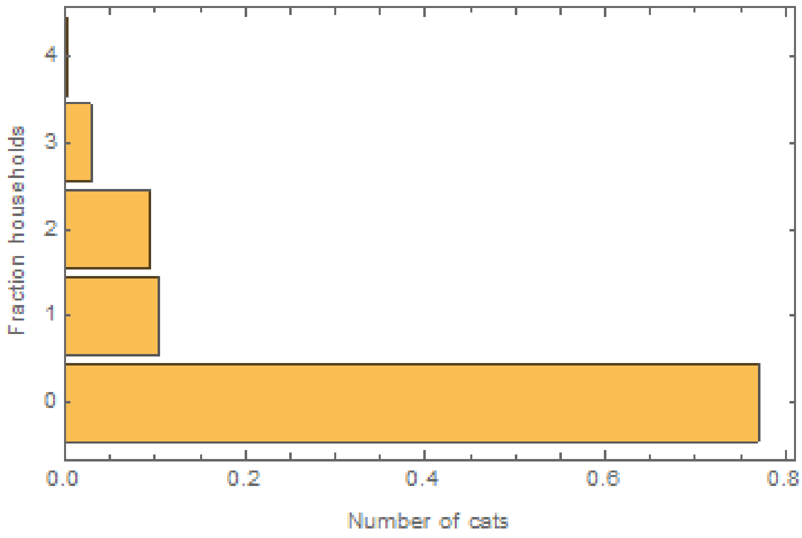

Figure 3 Distribution of cats inferred by considering that in 77% of the households no cats are present (0). The distribution of household with at least 1 cat was inferred by the probability of having k cats being $\binom{n}{k-1}p^{k-1}(1-p)^{n-k-1}$ *. The maximum number of cats was 4 and the average number of cats in a household with at least 1 cat was 1.7.*

3.3.5. Parameterization between-household transmission and scenarios

The extend of between household transmission by animals is unknown. Therefore we added an overall reduction parameter such that the overall R^* equalled the observed reproduction number observed on the first of May 2020 and that of 2021. This means that we assumed that the within-household transmission was stable in this period and the partial reproduction numbers equal to the ones estimated in this study, but the contacts between households was not. Furthermore this assumption means that the contacts between households is proportional over both humans and animals.

We compared the baseline reproduction number to the scenario when the transmission between humans and cats did not occur either outside the household, within the household or both. Here we assume that cats transmit the infection to other cats, but also to humans outside their household. The latter could occur due to contact outside a household, but also due to visitors of a household with an infected cat. The proportion ω should then be seen as the amount of time spend with individuals that do not live in the same household. The between-household reproduction number was calibrated by reducing all within-household reproduction numbers with a factor such that the R^* equalled the reproduction number at specific times during the SARS-CoV-2 pandemic in the Netherlands.

3.4. Results Between household transmission

Finally we included a what-if cats transmit the infection to humans and cats outside the own household by assuming that contact is made with individuals that do not live in the same household. The between-household reproduction number was calibrated by reducing the within-household transmission such that the R^* equalled the reproduction number as reported by the Dutch government (https://coronadashboard.rijksoverheid.nl/landelijk/reproductiegetal) at the first of March 2020 (1.83) and 2021 (1.06).

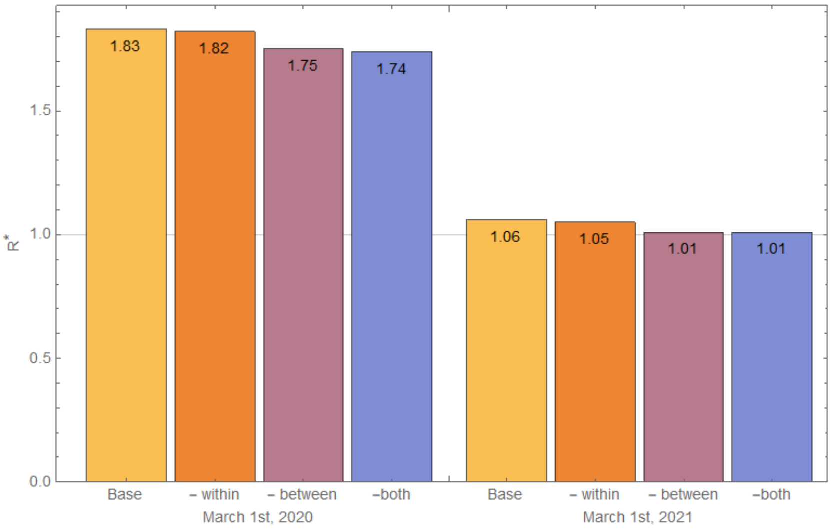

Figure 4 Reproduction numbers and the effect of removing cat contact within, between households or both, for two time-points in the epidemic (Base = calculation within and between household cat transmission, -within = no within-household cat transmission, -between = no between household cat transmission, -both= no cat transmission at all)

The effect of removing a route of transmission was less for within-household than between-household transmission of cats. The effect of within-household transmission is due to the decreased final size by additional transmission of the cats in the household after introduction of the infection. The contribution of between-household transmission lies in the increased number of households that can be infected.

4. References

1. de Jong MCM, Diekmann O, Heesterbeek JAP. How does transmission of infection depend on population size. In: Mollison D, editor. Epidemic Models: Their Structure and Relation to Data. Cambridge: Cambridge University Press; 1995. p. 84–94.

2. Diekmann O, Heesterbeek H, Britton T. Mathematical Tools for Understanding Infectious Disease Dynamics. Princeton University Press.; 2012. Chapter 12.

3. Ball F. A Unified Approach to the Distribution of Total Size and Total Area under the Trajectory of Infectives in Epidemic Models. Adv Appl Probab [Internet]. 1986;18(2):289–310. Available from: http://www.jstor.org/stable/1427301

4. Van Boven M, Koopmans M, Van Beest Holle MDR, Meijer A, Klinkenberg D, Donnelly CA, et al. Detecting emerging transmissibility of avian influenza virus in human households. PLoS Comput Biol. 2007;3(7):1394–402.

5. Reukers DFM, van Boven M, Meijer A, Rots N, Reusken C, Roof I, et al. High infection secondary attack rates of SARS-CoV-2 in Dutch households revealed by dense sampling. Clin Infect Dis. 2021;

6. Hall R, Becker NG. Preventing epidemics in a community of households. Epidemiol Infect [Internet]. 1996;117(3):443–55. Available from: http://www.pubmedcentral.nih.gov/articlerender.fcgi?artid=2271632&tool=pmcentrez&rendertype=abstract

7. Ball F, Lyne O. Optimal vaccination schemes for epidemics among a population of households, with application to variola minor in Brazil. Stat Methods Med Res [Internet]. 2006;15(5):481–97. Available from:

http://smm.sagepub.com/content/15/5/481.abstract%5Cnhttp://web.ebscohost.com/ehost/pdfviewer/pdfviewer?vid=2&hid=8&sid=ce603e78-8eff-4373-b5f7-3d27e7f839ba@sessionmgr13

8. Becker. NG. Modeling to Inform Infectious Disease Control. Chapman and Hall/CRC; 2015.

9. Werkgroep Feiten en Cijfers, van Heijst BAC, de Kort MAC, Overgaauw PAM, C.M. V, Beekmans MHC. Feiten & Cijfers Gezelschapsdierensector 2015 [Internet]. Den Bosch; 2015. Available from: https://www.rijksoverheid.nl/documenten/rapporten/2015/11/03/feiten-cijfers-gezelschapsdierensector-2015

288
