# Supplementary material for: Contribution of cats and dogs to SARS-CoV-2 transmission in households
Source: Front Vet Sci. 2023 Jul 14;10:1151772. doi: 10.3389/fvets.2023.1151772 (PMC10375487; doi:10.3389/fvets.2023.1151772)
Supplement: Supplementary file 2 [file Presentation_1.PDF]

# Household-structured model for SARS-CoV2 in humans and pets

Estimation of NGM within-households and calculation of R* in a household structured population

## Settings

## User defined generic functions

## Methods: Final size calculation and NGM estimation procedures

## Methods: Household structured population R*

# Load data

## Data Dutch survey among Covid-patients

In[∘]:=
```
ClearAll[surveydata]
surveydata = Import[inputdatadir <> "Data\\HH_cats_dogs_196_households_20210607.csv"];
surveydata[[1]]
colNcats = Position[surveydata[[1]], "n_cats_hh"][[1]];
If[#[[1]] == 0, "dog", If[#[[2]] == 0, "cat", "catdog"]] & /@
  surveydata[[2 ;; -1, {3, 4}]];
Tally[%]
(*use only the full data*)
nofdat = Length[surveydata[[2 ;; -1, Position[surveydata, "n_hum_hh"][[1, 2]]]]];
inputdata =
  {surveydata[[2 ;; -1, Position[surveydata, "n_hum_hh"][[1, 2]]]] - 1, (*s1*)
   surveydata[[2 ;; -1, Position[surveydata, "n_cats_hh"][[1, 2]]]], (*s2 if cat*)
   surveydata[[2 ;; -1, Position[surveydata, "n_dogs_hh"][[1, 2]]]], (*s2 if dog*)
   surveydata[[2 ;; -1,
    Position[surveydata, "n_animals_test"][[1, 2]]]], (*s2 if animal*)
   ConstantArray[1, nofdat], (*i1*)
   ConstantArray[0, nofdat], (*i2*)
   ((surveydata[[2 ;; -1, Position[surveydata, "n_hum_pos"][[1, 2]]]] /. "NA" → 0) - 1),
   (*c1*)
   surveydata[[2 ;; -1, Position[surveydata, "n_cats_SARS2pos"][[1, 2]]]],
   (*c2 if cat*)
   surveydata[[2 ;; -1, Position[surveydata, "n_dogs_SARS2pos"][[1, 2]]]],
   (*c2 if dog*)
   surveydata[[2 ;; -1, Position[surveydata, "n_animals_SARS2pos"][[1, 2]]]],
   (*c2 if animal*)
   surveydata[[2 ;; -1, Position[surveydata, "ID_HH"][[1, 2]]]]
  };
fullrecords = Transpose[Select[Transpose[inputdata], #[[1]] >= 0 &]];
fullrecordscatALL = fullrecords[[{1, 2, 5, 6, 7, 8}, All]] //. "NA" → 0;
fullrecordsdogALL = fullrecords[[{1, 3, 5, 6, 7, 9}, All]] //. "NA" → 0;
fullrecordspetALL = fullrecords[[{1, 4, 5, 6, 7, 10}, All]] //. "NA" → 0;
```

Out[∘]=
```
{ID_HH, n_animals_test, n_cats_hh, n_dogs_hh, n_animals_PCR_pos,
 n_animals_Seropos, n_animals_SARS2pos, n_cats_PCR_pos, n_cats_Seropos,
 n_cats_SARS2pos, n_dogs_PCR_pos, n_dogs_Seropos, n_dogs_SARS2pos, hh_cats_dogs,
 prop_animals_PCR_pos, prop_animals_Seropos, prop_animals_SARS2pos,
 prop_cats_PCR_pos, prop_cats_Seropos, prop_cats_SARS2pos, prop_dogs_PCR_pos,
 prop_dogs_Seropos, prop_dogs_SARS2pos, hum_pos_date1, n_hum_hh, n_hum_pos,
 hum_pos_date2, hum_pos_date3, hum_pos_date4, hum_pos_date5, hum_pos_date6}
```

Out[∘]=
```
{{dog, 98}, {cat, 73}, {catdog, 25}}
```

### Household-size distribution data

# Results: Estimation of parameters within household

## Descriptives

## Estimation of transmission parameters for the Dutch Survey

### Estimation of human to human transmission without estimating other transmission parameters

#### Per household

#### Compare models with non-susceptible hosts

In[ ]:=
```
(*select data with more than one human per household*)
petdata = Transpose[Select[Transpose[fullrecordspetALL], #[[1]] > 0 &]];
catdata = Transpose[Select[Transpose[fullrecordscatALL], #[[1]] > 0 &]];
dogdata = Transpose[Select[Transpose[fullrecordsdogALL], #[[1]] > 0 &]];
```

In[ ]:=
```
Print["R0 with number of animals as covariate"]
(*model for all data and linear relationship with number of animals as covariate*)
profPetLin = profile[{b}, {petdata[[1]], petdata[[3]], petdata[[5]]}, (petdata[[2]] - 1),
   petdata[[2]], {minb = -15, maxb = 15, db = 0.1, signif = 0.05, outprofile = False}];
Put[profPetLin, tempoutdir <> "profPetLin.txt"]
(*model for all data and linear relationship with number of cats as covariate*)
profCatLin = profile[{b}, {catdata[[1]], catdata[[3]], catdata[[5]]}, catdata[[2]],
   catdata[[2]], {minb = -5, maxb = 15, db = 0.01, signif = 0.05, outprofile = False}];
Put[profCatLin, tempoutdir <> "profCatLin.txt"]
(*model for all data and linear relationship with number of dogs as covariate*)
profDogLin = profile[{b}, {dogdata[[1]], dogdata[[3]], dogdata[[5]]}, dogdata[[2]],
   dogdata[[2]], {minb = -15, maxb = 15, db = 0.01, signif = 0.05, outprofile = False}];
Put[profDogLin, tempoutdir <> "profDogLin.txt"]
```

```
R0 with number of animals as covariate

Profile with non susceptible hosts

-15

15

Profile with non susceptible hosts

Out[•]= $Aborted

Profile with non susceptible hosts

Out[•]= $Aborted
```

```
Print["R0 with proportion of animals as covariate"]
(*model for all data and linear relationship
 with fraction animals of population as covariate*)
profPetProp = profile[{b}, {petdata[[1]], petdata[[3]], petdata[[5]]},
   (petdata[[2]] - 1) / (petdata[[1]] + petdata[[2]]),  petdata[[2]],
   {minb =  -15, maxb = 15, db = 0.1, signif = 0.05, outprofile = False}];
Put[profPetProp, tempoutdir <> "profPetProp.txt"]
(*model for all data and linear relationship
 with number of infected cats as covariate*)
profCatProp = profile[{b}, {catdata[[1]], catdata[[3]], catdata[[5]]},
   catdata[[2]] / (catdata[[1]] + catdata[[2]]), catdata[[2]],
   {minb = -15, maxb = 15, db = 0.01, signif = 0.05, outprofile = False}];
Put[profCatProp, tempoutdir <> "profCatProp.txt"]
(*model for all data and linear relationship
 with number of infected dogs as covariate*)
profDogProp = profile[{b}, {dogdata[[1]], dogdata[[3]], dogdata[[5]]},
   dogdata[[2]] / (dogdata[[1]] + dogdata[[2]]), dogdata[[2]],
   {minb = -15, maxb = 15, db = 0.01, signif = 0.05, outprofile = False}];
Put[profDogProp, tempoutdir <> "profDogProp.txt"]
```

In[ ]:=
```
Print["R0 with ratio of animals as covariate"]
(*model for all data and linear relationship
 with ratio petALLs of population as covariate*)
profPetRatio = profile[{b}, {petdata[[1]], petdata[[3]], petdata[[5]]},
   (petdata[[2]] - 1) / (petdata[[1]] + 1),  petdata[[2]],
   {minb = -15, maxb = 15, db = 0.1, signif = 0.05, outprofile = False}];
Put[profPetRatio, tempoutdir <> "profPetRatio.txt"]
(*model for all data and linear relationship
 with ratio animals of population as covariate*)
profCatRatio = profile[{b}, {catdata[[1]], catdata[[3]], catdata[[5]]},
   (catdata[[2]]) / (catdata[[1]] + 1),  catdata[[2]],
   {minb = -15, maxb = 15, db = 0.1, signif = 0.05, outprofile = False}];
Put[profCatRatio, tempoutdir <> "profCatRatio.txt"]
(*model for all data and linear relationship
 with ratio animals of population as covariate*)
profDogRatio = profile[{b}, {dogdata[[1]], dogdata[[3]], dogdata[[5]]},
   (dogdata[[2]]) / (dogdata[[1]] + 1),  dogdata[[2]],
   {minb = -15, maxb = 15, db = 0.05, signif = 0.05, outprofile = False}];
Put[profDogRatio, tempoutdir <> "profDogRatio.txt"]
```

```
R0 with ratio of animals as covariate

Profile with non susceptible hosts

-15

15

Profile with non susceptible hosts

-15

15

Profile with non susceptible hosts

-15

15
```

```
Print["Loading output"]
profPetLin = Get[tempoutdir <> "profPetLin.txt"];
profPetProp = Get[tempoutdir <> "profPetProp.txt"];
profPetRatio = Get[tempoutdir <> "profPetRatio.txt"];
profCatLin = Get[tempoutdir <> "profCatLin.txt"];
profCatProp = Get[tempoutdir <> "profCatProp.txt"];
profCatRatio = Get[tempoutdir <> "profCatRatio.txt"];
profDogLin = Get[tempoutdir <> "profDogLin.txt"];
profDogProp = Get[tempoutdir <> "profDogProp.txt"];
profDogRatio = Get[tempoutdir <> "profDogRatio.txt"];
```

Create table for publication

In[∘]:=
```
({{"Model", "R", "CI-ll", "CI-ul", "b1", "b1-ll", "b1-ul", "AIC"},
   firsttableRow["All data only R", profPetRatio],
   tableRow["Ratio pet/human ", profPetRatio],
   tableRow["Ratio cat/human ", profCatRatio],
   tableRow["Ratio dog/human ", profDogRatio]
  }) // TableForm
```

Out[∘]//TableForm=

| Model | R | CI-ll | CI-ul | b1 | b1-ll | b1-ul | AIC |
|---|---|---|---|---|---|---|---|
| All data only R | 1.17 | 0.93 | 1.47 | - | - | - | 362.87 |
| Ratio pet/human | 0.88 | 0.57 | 1.34 | 1.97 | 0.18 | 4.79 | 355.04 |
| Ratio cat/human | 0.91 | 0.57 | 1.39 | 1.19 | -0.05 | 2.97 | 358. |
| Ratio dog/human | 1.1 | 0.69 | 1.66 | 0.33 | -0.15 | 2.19 | 364.48 |

In[∘]:=
```
Histogram[{Select[HHR0dog[[All, {3, 5}]], #[[2]] < 200 &][[All, 1]],
   Select[HHR0dog[[All, {3, 5}]], #[[2]] > 200 &][[All, 1]]},
  Automatic, "Probability", Frame → True, ChartLegends → {R0 < ∞, R0 == ∞},
  FrameLabel → { "Proportion dogs", "Fraction households"}]
```

Out[∘]=

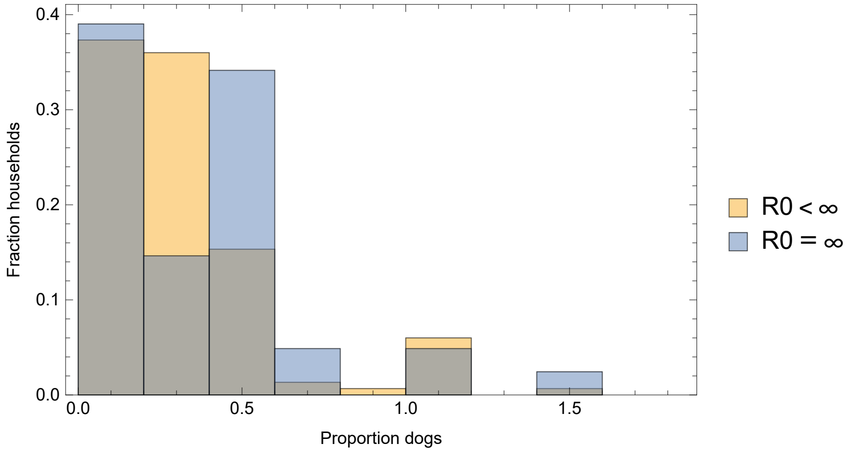


# Estimation of companion animals and humans

## Estimation and profile likelihoods

```
ClearAll[R, estRpet, profileR11pet, profileR21pet, profileR12pet, profileR22pet]
```

```
Off[FindMinimum::reged] (*turn of this warning as it is clear from the estimates.*)
(*point estimate*)
Print["Estimation of " <> scenario]
estRpet = FindMaximum[Log[l[R11, R21, R12, R22,
     {fullrecordspetALL[[1]], fullrecordspetALL[[2]], fullrecordspetALL[[3]],
      fullrecordspetALL[[4]], fullrecordspetALL[[5]], fullrecordspetALL[[6]]}]],
   {R11, 0.2, 0.001, 200}, {R21, 0.2, 0.0, 200}, {R12, 0.2, 0.0001, 200},
   {R22, .2, 0., 200}, AccuracyGoal → 6];
Print["Estimation of " <> scenario <> " Scenario"]
Print["Optimal {R11,R21,R12,R22} = ", {R11, R21, R12, R22} /. estRpet[[2]]];
Print["Log-likelihood: ", estRpet[[1]]];
Put[estRpet, tempoutdir <> "estRpet" <> scenario <> ".txt"]
```

Estimation of Ratio

FindMinimum: The point {1.19343, 0.384955, 0.630241, 0.} is at the edge of the search region {0., 200.} in coordinate 4 and the computed search direction points outside the region.

Estimation of Ratio Scenario

Optimal {R11,R21,R12,R22} = {1.19343, 0.384955, 0.630241, 0.}

Log-likelihood: -285.891

In[ ]:=
```
(*determine profile likelihoods*)
profileR11pet =
  Table[{R11, FindMaximum[Log[l[R11, R21, R12, R22, {fullrecordspetALL[[1]],
         fullrecordspetALL[[2]], fullrecordspetALL[[3]], fullrecordspetALL[[4]],
         fullrecordspetALL[[5]], fullrecordspetALL[[6]]}]], {R21, 0.2, 0.0, 200},
     {R12, 0.2, 0.0001, 200}, {R22, .2, 0, 200}, AccuracyGoal → 6]}, {R11, 0.5, 2.5, 0.1}];
Put[profileR11pet, tempoutdir <> "profileR11pet" <> scenario <> ".txt"]
ci[profileR11pet, estRpet, 0.05]
```

FindMaximum: The line search decreased the step size to within the tolerance specified by AccuracyGoal and PrecisionGoal but was unable to find a sufficient increase in the function. You may need more than MachinePrecision digits of working precision to meet these tolerances.

Out[ ]= {ll → 0.899825, ul → 1.43716}

In[ ]:=
```
profileR21pet = Table[{R21, FindMaximum[Log[l[R11, R21, R12, R22,
        {fullrecordspetALL[[1]], fullrecordspetALL[[2]], fullrecordspetALL[[3]],
         fullrecordspetALL[[4]], fullrecordspetALL[[5]], fullrecordspetALL[[6]]}]],
     {R11, 0.2, 0.001, 200}, {R12, 0.2, 0.0001, 200}, {R22, .2, 0, 200}, AccuracyGoal → 6]},
   {R21, {0.0001, 0.01}~Join~Table[i, {i, 0.25, 5.5, .5}]}];
Put[profileR21pet, tempoutdir <> "profileR21pet" <> scenario <> ".txt"]
ci[profileR21pet, estRpet, 0.05]
```

FindMaximum: The line search decreased the step size to within the tolerance specified by AccuracyGoal and PrecisionGoal but was unable to find a sufficient increase in the function. You may need more than MachinePrecision digits of working precision to meet these tolerances.

Out[ ]= {ll → -∞, ul → 2.15019}

In[∘]:=
```
profileR12pet = Table[{R12, FindMaximum[Log[l[R11, R21, R12, R22,
      {fullrecordspetALL[[1]], fullrecordspetALL[[2]], fullrecordspetALL[[3]],
       fullrecordspetALL[[4]], fullrecordspetALL[[5]], fullrecordspetALL[[6]]}]],
    {R11, 0.2, 0.001, 200}, {R21, 0.2, 0.0, 200}, {R22, .2, 0, 200}, AccuracyGoal → 6]},
   {R12, Table[j, {j, {0.001, .1, 0.25}~Join~Table[i, {i, 0.3, 1.0, 0.1}]}]}];
Put[profileR12pet, tempoutdir <> "profileR12pet" <> scenario <> ".txt"]
ci[profileR12pet, estRpet, 0.05]
```

FindMaximum: The line search decreased the step size to within the tolerance specified by AccuracyGoal and PrecisionGoal but was unable to find a sufficient increase in the function. You may need more than MachinePrecision digits of working precision to meet these tolerances.

Out[∘]= {ll → 0.41774, ul → 0.76556}

In[∘]:=
```
profileR22pet = Table[{R22, FindMaximum[Log[l[R11, R21, R12, R22,
      {fullrecordspetALL[[1]], fullrecordspetALL[[2]], fullrecordspetALL[[3]],
       fullrecordspetALL[[4]], fullrecordspetALL[[5]], fullrecordspetALL[[6]]}]],
    {R11, 0.2, 0.01, 20}, {R21, 0.2, 0.0, 20}, {R12, 0.2, 0.01, 20}, AccuracyGoal → 6]},
   {R22, Table[j, {j, {0.00, 0.05, 0.1, .15, .175}~Join~Table[i, {i, 0.25, 2.5, 0.25}]}]}];
Put[profileR22pet, tempoutdir <> "profileR22pet" <> scenario <> ".txt"]
ci[profileR22pet, estRpet, 0.05]
```

Out[∘]= {ll → -∞, ul → 0.274222}

In[∘]:=
```
On[FindMinimum::reged] (*turn of this warning as it is clear from the estimates.*)
```

Bootstrap for Ratio

Doing 100 bootstraps.

## Bootstrap values of R

## Load estimation, profile likelihoods and bootstrap and visualize/ tabelize

In[∘]:=
```
Print["Loading Scenario:" <> scenario]
estRpet = Get[tempoutdir <> "estRpet" <> scenario <> ".txt"];
```

Loading Scenario:Ratio

In[∘]:=
```
(*load previously saved output*)
profileR11pet = Get[tempoutdir <> "profileR11pet" <> scenario <> ".txt"];
profileR21pet = Get[tempoutdir <> "profileR21pet" <> scenario <> ".txt"];
profileR12pet = Get[tempoutdir <> "profileR12pet" <> scenario <> ".txt"];
profileR22pet = Get[tempoutdir <> "profileR22pet" <> scenario <> ".txt"];
bootstrapsRpet = Get[tempoutdir <> "bootstrapsRpet" <> scenario <> ".txt"];
```

In[ ]:=
```
estRpet;
ciR11pet = ci[profileR11pet, estRpet, 0.05];
ciR21pet = ci[profileR21pet, estRpet, 0.05];
ciR12pet = ci[profileR12pet, estRpet, 0.05];
ciR22pet = ci[profileR22pet, estRpet, 0.05];
MapThread[Flatten[Round[{#1[[2]], {ll, ul} /. #2}, .001]] &,
  {estRpet[[2]], {ciR11pet, ciR21pet, ciR12pet, ciR22pet} }];
{{"pet", "Estimate", "95%-CI", ""}} ~ Join ~
   MapThread[Prepend, {%, {"R11", "R21", "R12", "R22"}}] // TableForm;
(% /. -∞ → "Undet.") // TableForm
```

Out[ ]//TableForm=

| pet | Estimate | 95%-CI | |
|---|---|---|---|
| R11 | 1.193 | 0.9 | 1.437 |
| R21 | 0.385 | Undet. | 2.15 |
| R12 | 0.63 | 0.418 | 0.766 |
| R22 | 0. | Undet. | 0.274 |

In[ ]:=
```
plotR0 =.;
Print["Plotting Scenario: " <> scenario]

bstrpRpet = bootstrapRvalues[#] & /@ bootstrapsRpet;
```

Plotting Scenario: Ratio

In[ ]:=
```
bstrpRpetCIq =
  Function[data, Select[data, #[[2]] ≤ Quantile[data[[All, 2]], 0.975] && #[[2]] ≥ Quantile[
        data[[All, 2]], 0.025] &]] /@ SplitBy[Sort[Flatten[bstrpRpet, 1]] , First];
llRpets = Flatten[Function[x, Select[x, #[[2]] == Min[x[[All, 2]]] &]][Union[#]] & /@
   bstrpRpetCIq, 1]
ulRpets = Flatten[Function[x, Select[x, #[[2]] == Max[x[[All, 2]]] &]][Union[#]] & /@
   bstrpRpetCIq, 1]
```

Out[ ]= $\{\{\frac{1}{6}, 0.996887\}, \{\frac{1}{5}, 0.994387\}, \{\frac{1}{4}, 1.00978\}, \{\frac{1}{3}, 1.03062\}, \{\frac{2}{5}, 1.0434\}, \{\frac{1}{2}, 1.05859\}, \{\frac{3}{5}, 1.08882\}, \{\frac{2}{3}, 1.09129\}, \{\frac{3}{4}, 1.10696\}, \{\frac{4}{5}, 1.11404\}, \{1, 1.13313\}, \{\frac{4}{3}, 1.18268\}, \{\frac{3}{2}, 1.19734\}, \{\frac{5}{3}, 1.22293\}, \{2, 1.26001\}, \{3, 1.36057\}, \{6, 1.58639\}\}$

Out[ ]= $\{\{\frac{1}{6}, 1.45206\}, \{\frac{1}{5}, 1.4611\}, \{\frac{1}{4}, 1.4712\}, \{\frac{1}{3}, 1.48499\}, \{\frac{2}{5}, 1.49104\}, \{\frac{1}{2}, 1.51238\}, \{\frac{3}{5}, 1.53911\}, \{\frac{2}{3}, 1.54522\}, \{\frac{3}{4}, 1.5596\}, \{\frac{4}{5}, 1.57396\}, \{1, 1.59495\}, \{\frac{4}{3}, 1.64284\}, \{\frac{3}{2}, 1.67247\}, \{\frac{5}{3}, 1.717\}, \{2, 1.74654\}, \{3, 1.87633\}, \{6, 2.22977\}\}$

In[ ]:=
```
(calcR0[ratio, scenario] /. estRpet[[2]]) /. ratio → 6
```

Out[ ]= 1.94273

In[ ]:=
```
plotR0 = calcR0[ratio, scenario];
plotPEfull = Plot[{Evaluate[plotR0 /. estRpet[[2]]]},
   {ratio, 1/6, 6.0}, PlotRange → {{-.1, 6.5}, {-.1, 2.5}}, Frame → True,
   FrameLabel → {"pet to human ratio", "Household R / Fraction of households"},
   GridLines → {None, {1}}];
```

In[ ]:=
```
bootstrapplot = ListLinePlot[{llRpets, ulRpets}, PlotRange → {0, All}, PlotStyle → None,
   GridLines → {None, {1}}, FillingStyle → Directive[Gray, Opacity[0.2]],
   Filling → {{1 → {2}}}, Frame → True, FrameLabel →
    {"Companion animal to human ratio", "Household R / Fraction of households"}];
Show[ bootstrapplot, plotPEfull, PlotRange → {{-.1, 6.5}, {-.1, 3}}];
 Histogram[(fullrecordspetALL[[2]] + fullrecordspetALL[[4]]) /
   (fullrecordspetALL[[1]] + fullrecordspetALL[[3]]), Automatic, "Probability"];
plotRpet = Show[ bootstrapplot, plotPEfull, %, PlotRange → {{-.1, 6.5}, {-.1, 3}}]
Export[tempoutdir <> "petR0plotCI" <> scenario <> ".jpg", plotRpet ];
```

Out[ ]=

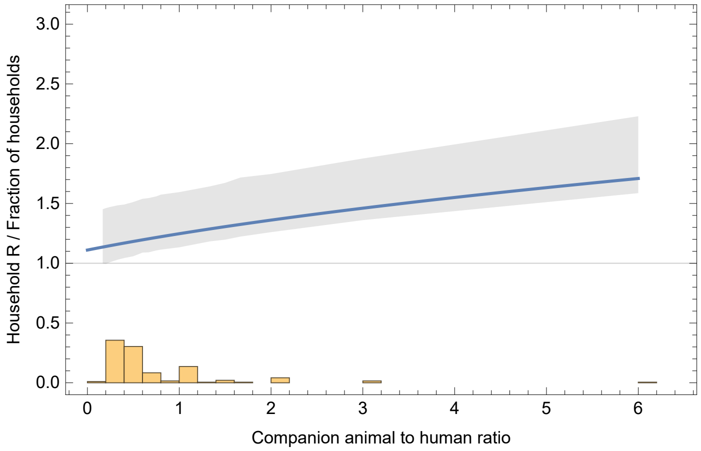


# Estimation of cats and humans

## Estimation and profile likelihoods

## Bootstrap values of R

## Load estimation, profile likelihoods and bootstrap and visualize/ tabelize

In[ ]:=
```
Print["Loading Scenario:" <> scenario]
estRcat  = Get[tempoutdir <> "estRcat" <> scenario <> ".txt"];
```

```
Loading Scenario:Ratio
```

In[•]:=
```
(*load previously saved output*)
profileR11cat = Get[tempoutdir <> "profileR11cat" <> scenario <> ".txt"];
profileR21cat = Get[tempoutdir <> "profileR21cat" <> scenario <> ".txt"];
profileR12cat = Get[tempoutdir <> "profileR12cat" <> scenario <> ".txt"];
profileR22cat = Get[tempoutdir <> "profileR22cat" <> scenario <> ".txt"];
bootstrapsRcat = Get[tempoutdir <> "bootstrapsRcat" <> scenario <> ".txt"];
```

In[•]:=
```
ciR11cat = ci[profileR11cat, estRcat, 0.05];
ciR21cat = ci[profileR21cat, estRcat, 0.05];
ciR12cat = ci[profileR12cat, estRcat, 0.05];
ciR22cat = ci[profileR22cat, estRcat, 0.05];
(*Create a table with estimates of parameters and confidence intervals*)
MapThread[Flatten[Round[{#1[[2]], {ll, ul} /. #2}, .001]] &,
  {estRcat[[2]], {ciR11cat, ciR21cat, ciR12cat, ciR22cat} }];
{{"cat", "Estimate", "95%-CI", ""}} ~ Join ~
   MapThread[Prepend, {%, {"R11", "R21", "R12", "R22"}}] // TableForm;
(% /. -∞ → "Undet.") // TableForm
```

Out[•]//TableForm=

| cat | Estimate | 95%-CI | |
|---|---|---|---|
| R11 | 1.257 | 0.923 | 1.485 |
| R21 | 0.301 | Undet. | 2.02 |
| R12 | 0.556 | 0.357 | 0.777 |
| R22 | 0. | Undet. | 0.483 |

In[•]:=
```
(*Get bootstrap values and confidence intervals of R*)
bstrpRcat = bootstrapRvalues[#] & /@ bootstrapsRcat;
bstrpRcatCIq =
  Function[data, Select[data, #[[2]] ≤ Quantile[data[[All, 2]], 0.975] && #[[2]] ≥ Quantile[
        data[[All, 2]], 0.025] &]] /@ SplitBy[Sort[Flatten[bstrpRcat, 1]] , First];
(*Get lower and upper limit of confidence interval*)
llRcats =
 Flatten[Function[x, Select[x, #[[2]] == Min[x[[All, 2]]] &]][Union[#]] & /@ bstrpRcatCIq, 1]
ulRcats = Flatten[
  Function[x, Select[x, #[[2]] == Max[x[[All, 2]]] &]][Union[#]] & /@ bstrpRcatCIq, 1]
```

Out[•]= $\{\{0, 0.9852\}, \{\frac{1}{5}, 1.00215\}, \{\frac{1}{4}, 1.01409\}, \{\frac{1}{3}, 1.01539\}, \{\frac{2}{5}, 1.02674\}, \{\frac{1}{2}, 1.02966\}, \{\frac{3}{5}, 1.04753\}, \{\frac{2}{3}, 1.0523\}, \{\frac{3}{4}, 1.06265\}, \{1, 1.07316\}, \{\frac{3}{2}, 1.10688\}, \{2, 1.13264\}, \{3, 1.28507\}\}$

Out[•]= $\{\{0, 1.48536\}, \{\frac{1}{5}, 1.4998\}, \{\frac{1}{4}, 1.5185\}, \{\frac{1}{3}, 1.53066\}, \{\frac{2}{5}, 1.5318\}, \{\frac{1}{2}, 1.54959\}, \{\frac{3}{5}, 1.57758\}, \{\frac{2}{3}, 1.56907\}, \{\frac{3}{4}, 1.56059\}, \{1, 1.61185\}, \{\frac{3}{2}, 1.67813\}, \{2, 1.74329\}, \{3, 1.8613\}\}$

In[ ]:=
```
Print["Plotting Scenario: " <> scenario]
plotR0 = calcR0[ratio, scenario];
(*Create a plot of the point estimate of R*)
plotPEfull = Plot[{Evaluate[plotR0 /. estRcat[[2]]]},
   {ratio, 0, 3.}, PlotRange → {{-0.1, 6.5}, {-.1, 2.5}}, Frame → True,
   FrameLabel → {"Cat to human ratio", "Household R / Fraction of households"},
   GridLines → {None, {1}}];
```

```
Plotting Scenario: Ratio
```

In[ ]:=
```
calcR0[ratio, scenario] /. {R11 → 1.257, R21 → 0.301, R12 → 0.556, R22 → 0, ratio → 3}
```

Out[ ]=
```
1.57564
```

In[ ]:=
```
(*Plot*)
bootstrapplot =
  ListLinePlot[{llRcats, ulRcats}, PlotRange → {{-.1, 6.5}, {-.1, 2.5}}, PlotStyle → None,
   FillingStyle → Directive[Gray, Opacity[0.2]], Filling → {{1 → {2}}}, Frame → True,
   FrameLabel → {"Cat to human ratio", "Household R / Fraction of households"},
   GridLines → {None, {1}}];
```

In[ ]:=
```
Show[ bootstrapplot, plotPEfull, PlotRange → {{-.1, 6.5}, {-.1, 2.5}}];
Histogram[(fullrecordscatALL[[2]] + fullrecordscatALL[[4]]) /
   (fullrecordscatALL[[1]] + fullrecordscatALL[[3]]), Automatic, "Probability"];
plotCI = Show[ {bootstrapplot, plotPEfull, %}]
Export[tempoutdir <> "catR0plotCI" <> scenario <> ".jpg", plotCI];
```

Out[ ]=

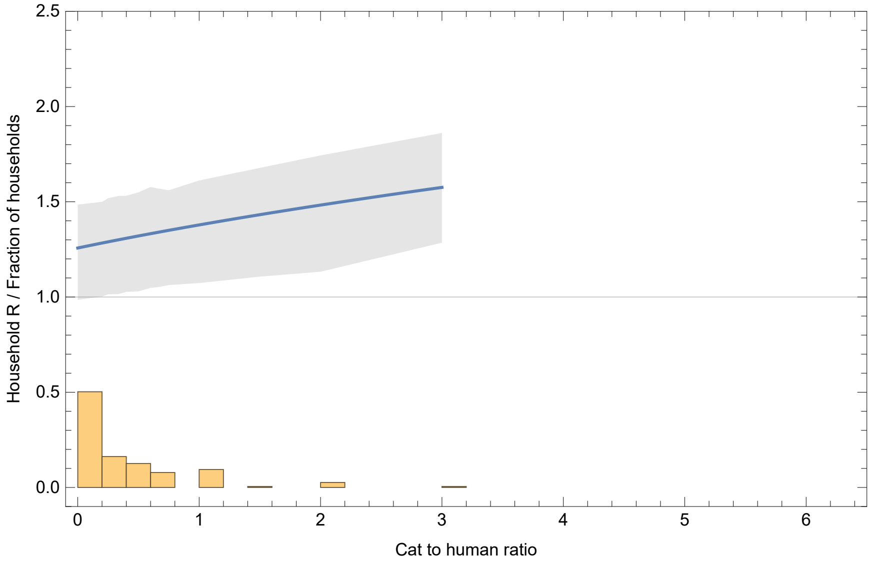


## Estimation of dogs and humans

### Estimation and profile likelihoods

### Bootstrap values of R

#### Load estimation, profile likelihoods and bootstrap and visualize/ tabelize

## Sensitivity analysis for estimation of transmission parameters for the Dutch Survey

# Results: household structured population

## Household size distribution

## Scenarios
